# Seed quality as a proxy of climate-ready orphan legumes: the need for a multidisciplinary and multi-actor vision

**DOI:** 10.3389/fpls.2024.1388866

**Published:** 2024-08-01

**Authors:** Alma Balestrazzi, Cinzia Calvio, Anca Macovei, Andrea Pagano, Patrick Laux, Hassane Moutahir, Loїc Rajjou, Eleni Tani, Dimosthenis Chachalis, Christos Katsis, Lamiae Ghaouti, Said Gmouh, Sanaa Majid, Amine Elleuch, Moez Hanin, Bassem Khemakhem, Hanen El Abed, Joao Nunes, Susana Araújo, Aziz Benhamrouche, Mohand Bersi

**Affiliations:** ^1^ Department of Biology and Biotechnology “L. Spallanzani”, University of Pavia, Pavia, Italy; ^2^ Institute of Meteorology and Climate Research (IMK-IFU), Karlsruhe Institute of Technology, Garmisch-Partenkirchen, Germany; ^3^ Université Paris-Saclay, National Research Institute for Agriculture, Food and the Environment (INRAE), AgroParisTech, Institut Jean-Pierre Bourgin (IJPB), Versailles, France; ^4^ Laboratory of Plant Breeding and Biometry, Department of Crop Science, Agricultural University of Athens, Athens, Greece; ^5^ Department of Pesticides’ Control and Phytopharmacy, Benaki Phytopathological Institute, Athens, Greece; ^6^ Agroland S.A., Sofades, Greece; ^7^ Department of Plant Production, Protection and Biotechnology, Hassan II Institute of Agronomy and Veterinary Medicine, Rabat, Morocco; ^8^ Laboratory Laboratory of Engineering and Materials (LIMAT), Faculty of Sciences Ben M’sick, University Hassan II of Casablanca, Casablanca, Morocco; ^9^ Laboratory GeMEV, Faculty of Sciences Aïn Chock, University Hassan II of Casablanca, Casablanca, Morocco; ^10^ Higher Institute of Biotechnology, University of Sfax, Sfax, Tunisia; ^11^ Center Bio R&D Unit, Association BLC3–Technology and Innovation Campus, Oliveira do Hospital, Portugal; ^12^ Institute of Architecture and Earth Science, University Ferhat Abbas-Setif 1, Setif, Algeria

**Keywords:** climate change, Greater Mediterranean Region, resilience, food security, multi-actor approach, underutilized legumes, seed priming and seed system, vulnerability mapping

## Abstract

In developing countries, orphan legumes stand at the forefront in the struggle against climate change. Their high nutrient value is crucial in malnutrition and chronic diseases prevention. However, as the ‘orphan’ definition suggests, their seed systems are still underestimated and seed production is scanty. Seed priming is an effective, sustainable strategy to boost seed quality in orphan legumes for which up-to-date guidelines are required to guarantee reliable and reproducible results. How far are we along this path? What do we expect from seed priming? This brings to other relevant questions. What is the socio-economic relevance of orphan legumes in the Mediterranean Basin? How to potentiate a broader cultivation in specific regions? The case study of the BENEFIT-Med (Boosting technologies of orphan legumes towards resilient farming systems) project, developed by multidisciplinary research networks, envisions a roadmap for producing new knowledge and innovative technologies to improve seed productivity through priming, with the long-term objective of promoting sustainability and food security for/in the climate-sensitive regions. This review highlights the existing drawbacks that must be overcome before orphan legumes could reach the state of ‘climate-ready crops’. Only by the integration of knowledge in seed biology, technology and agronomy, the barrier existing between research bench and local agricultural fields may be overcome, generating high-impact technical innovations for orphan legumes. We intend to provide a powerful message to encourage future research in line with the United Nations Agenda 2030 for Sustainable Development.

## 1 Introduction

In the climate-vulnerable regions, local populations are exposed to high mortality rate due to malnutrition exacerbated by climate change. The complex interactions between climate-change and food systems, and their domino effect on human health, represent a major challenge that requires urgent and enduring actions (Hertel et al., 2021; Zurek et al., 2022). Mitigation strategies have been designed to speed up the transition towards sustainable, climate-resilient food systems, e.g. those reported by the Intergovernmental Panel on Climate Change: demand-side changes, protection of ecosystems, improved farm management, and decarbonizing supply chain (IPCC, 2022). Mitigation strategies involve multi-level players, such as political, financial, industrial, and scientific actors, together with the civil society (IPCC, 2022; Global Panel on Agriculture and Food Systems for Nutrition, 2023).

Major crops show poor genetic diversity and high vulnerability to extreme climate events, implying their replacement by a new generation of ‘climate-ready’ crops (Bourke et al., 2021; Xiong et al., 2022). This goal can be achieved by valorizing local minor crops (orphan crops or neglected, underutilized species-NUS) that currently have very limited impact on the market but are yet essential for small farmers’ survival in developing countries (Tadele, 2019; Popoola et al., 2022). According to FAO (2019), these species “*have lost relevance over the last 500 years due to societal, agronomic, or biological factors*”. Orphan crops include legumes, appreciated for their drought tolerance and capacity to improve soil fertility (Popoola et al., 2022), on top of their nutritional and medicinal features (Ogbole et al., 2023). These species and their seed systems are still undervalued, despite the crucial role they play in supporting human and animal nutrition, and favoring the growth of other crops (Cullis and Kunert, 2017; Popoola et al., 2022). This work provides a multidisciplinary and collaborative vision for the creation of new solutions allowing to cope with the challenges related to orphan legumes, with a particular focus on seed quality issues.

## 2 Orphan legumes as ‘climate-ready’ crops

Major crops are not able to adapt to climate change. Conversely, orphan legumes are ‘climate-smart’ plants with outstanding agronomic features, able to support sustainable livelihood, even under harsh environments (Popoola et al., 2022). Orphan legumes did not undergo the intensive breeding selection experienced by major crops, thus retaining a huge genetic variability and unique assortments of stress resilience traits. These enormous genetic resources are exposed to erosion, linked to their underutilization, reducing the chances of their future exploitation (Popoola et al., 2022; Kumar et al., 2023). Orphan legumes are poorly represented in germplasm collections and lack formal seed supply systems. They are usually identified based on their local relevance, adaptation to marginal areas, agroecological niches. Their cultivation and use are still relying on local knowledge. The limited market potential and lack of functional value chains hamper full valorization. To revert this status, several actions are needed at different levels: policies should encourage basic/applied research on these species, breeding programs should be established to obtain improved hybrids, agrifood industries should diversify their investments including orphan legumes, and an increased consumers’ awareness should stimulate the process from the basis, laying at the intersection between supply and demand (Cullis et al., 2019; Borelli et al., 2020; Elolu et al., 2023).

Orphan legumes are native to Africa, Asia, South America, North America, South Pacific, and Australia, although some species are classified as orphan in one location and as conventional crops in other areas (Talabi et al., 2022). Institutions involved in the identification and the proper management of orphan legumes are shown in **Table 1**. A list of underutilized crops (Future Smart Food) was compiled by the African Orphan Crops Consortium (AOCC) and the Agriculture Organization of the United Nations Regional Office for Asia and the Pacific (FAO/RAP), according to high nutritional profile, climate, resilience, local availability, and economic viability.

**Table 1:** List of International Istitutions that currently host germplasm collections of orphan legumes.

Host Institution	Underutilized Species	Reference
CIGIAR https://www.cgiar.org/ AOOC https://africanorphancrops.org/ FAO/RAP https://www.fao.org/asiapacific/en/ CROP TRUST https://www.croptrust.org/ CFF https://cropsforthefutureuk.org/ IITA https://www.iita.org/ WVC https://avrdc.org/ PGRCU-USDA-ARS https://www.ars.usda.gov/southeast-area/griffin-ga/pgrcu/docs/about-pgrcu/	African yam bean (*Sphenostylis stenocarpa* L.) African winged bean (*Psophocarpus tetragonolobus* L.) Moth bean (*Vigna aconitifolia* L.) Bambara groundnut (*Vigna subterranean* L.) cowpea (*Vigna unguiculata* L.) Kersting’s groundnut (*Macrotyloma geocarpum* L.) Lima bean (*Phaseolus lunatus* L.) Sword bean (*Canavalia gladiata* L.) Jack bean (*Canavalia ensiformis* L.) hyacinth bean (*Lablab purpureus* L.) pigeon pea (*Cajanus cajan* L.) ghaf (*Prosopis cineraria* L.) gum acacia (*Acacia senegal* L.) cluster bean (*Cyamopsis tetragonoloba* L.) Trigonella (*Trigonella foenum-graecum* L.) grass pea (*Lathyrus sativus* L.) forage pea (*Pisum sativum* var. arvense)	Li and Siddique, 2018 Hendre et al., 2019 Talabi et al., 2022 Popoola et al., 2022 Abberton et al., 2022 Joshi et al., 2023 Vaz Patto and Rubiales, 2014 Powers and Thavarajah, 2019 Visuvanathan et al., 2022 Ramya et al., 2022 Gonçalves et al., 2022 Greveniotis et al., 2022

CGIAR, Consultative Group for International Agricultural Research; AOCC, African Orphan Crops Consortium; FAO/RAP, Agriculture Organization of the United Nations Regional Office for Asia and the Pacific; CFF, Crops for the Future; IITA, International Institute of Tropical Agriculture, Nigeria; WVC, World Vegetable Center, Taiwan; PGRCU-USDA-ARS, Plant Genetic Resources Conservation Unit - U. S. Department of Agriculture - Agricultural Research Service.

## 3 How would seed quality impact the productivity of orphan legumes under the challenges of climate change?

### 3.1 Seed systems: a variegated landscape moving towards climate-resilience

**Figure 1:**
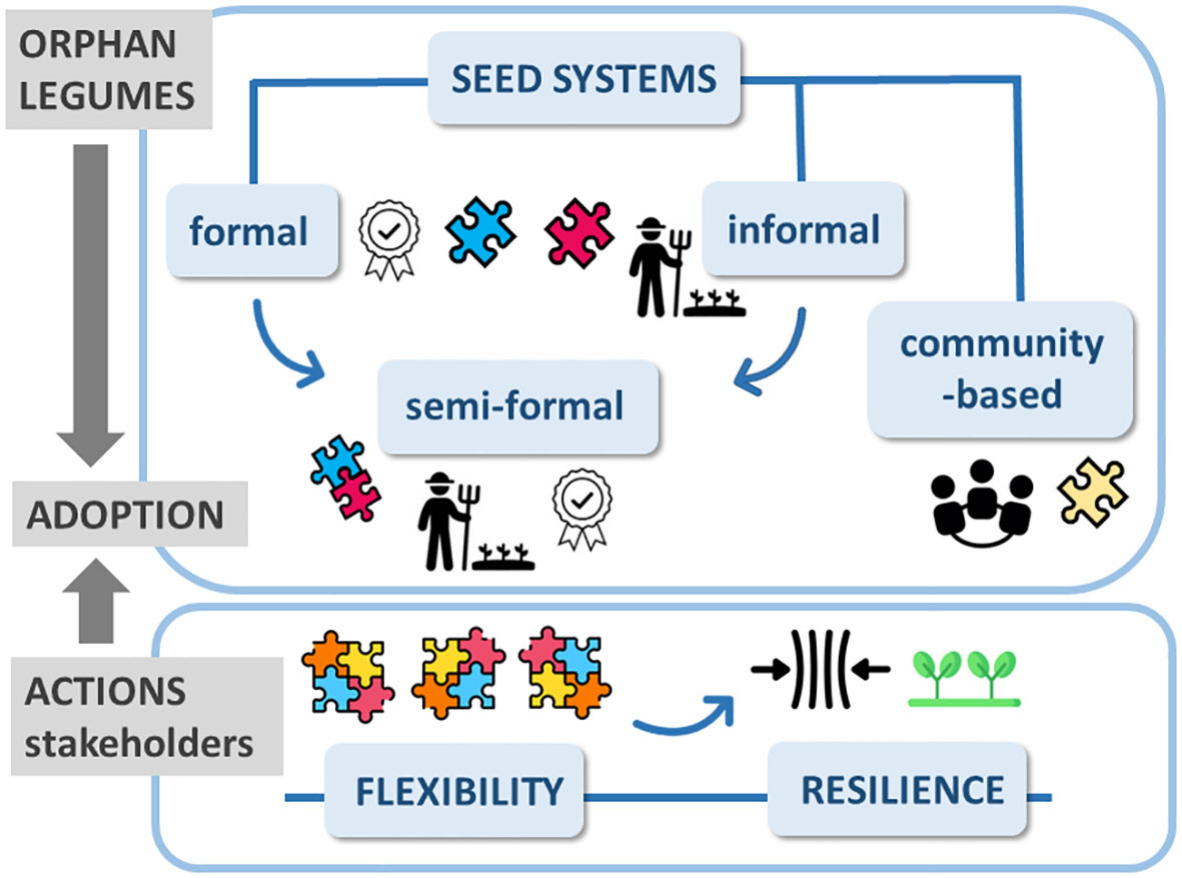
Seed systems are crucial vehicles that allow farmers to acquire good quality seeds of new varieties, playing an essential role in the process of orphan legumes adoption in the climate-vulnerable regions of Mediterranean area. They are part of a variegated puzzle, since different types of seed systems currently exist: formal, semi-formal, informal, and community-based seed systems. Actions should be taken in order to promote remodelling and integration of the different seed systems, based on local agronomic, technical, socio-economic, and cultural needs. Stakeholders, including policy makers, are committed to provide such flexibility, as a key factor towards better climate resilience of local agroecosystems.

Enhanced seed vigor is the first step towards the valorization of any crop and, in particular, of those missing an official seed supply system. Seed systems include all the activities for variety development (Louwaars and Manicad, 2022). They are classified into categories that often co-exist in the same territory, resulting in a variegated landscape (**Figure 1**). *Community-based seed systems* rely on experienced farmers who take care of seeds provided by other farmers and share the improved products (Gonsalves, 2013). Besides a special care in ensuring a wide agrobiodiversity, the current challenges for community-based seed systems refer to a more inclusive approach aimed at engaging increased numbers of local farmers, the organization of wide and frequent participatory activities, as well as the development of strategic partnerships with public/private sectors. Community-based seed systems are the core of local enterprises (Vernooy et al., 2022). In *formal seed system*, activities are strictly regulated. Seed producers are registered and possess a certification under the control of public institutions. Certified seeds are also produced in collaboration with private seed companies (Kansiime et al., 2021). Small farmers still rely on *informal seed system* in which seeds are acquired through personal exchanges, gifts, local markets. However, informally traded seeds are at risk in terms of genetic purity, viability and productivity. Informal systems, the main source of seeds in climate-vulnerable regions, do not provide deal with ‘breeder seed’ (genetically and physically pure seeds propagated under strict control) and ‘basic seed’ (produced by registered growers, under the control of certification agencies) (Vernooy et al., 2022). Finally, *semi-formal seed systems* integrate the formal and informal systems. They are represented by farmers who manage all the value chain steps, reaching enhanced seed quality with the support of technical and financial partners (ICRISAT, 2008). In response to adverse environments, the different seed systems provide complementary characteristics (**Figure 1**), and seeds can be moved from the informal to the formal context (or vice versa) to buffer such changes (Louwaars and Manicad, 2022). However, this is not sufficient to mitigate the impact of severe climate change and ensure food security. The resilience of this variegated landscape should be reinforced not only in terms of scientific/technical skills, required for proper management of germplasm collections, but also by supporting socio-economic and political strategies. The goal is the design of political roadmaps to protect and provide local farmers with the most adapted varieties, in a rapid and efficient manner (Louwaars and Manicad, 2022).

### 3.2 Seed traits for climate-resilience

For the correct management/selection of the seeds to be used in challenging conditions, a fundamental asset is the identification of the most relevant seed traits in terms of climate resilience. Detailed agronomic and genetic information is not generally available for orphan legumes. Thus, it is imperative to link phenotype- and genotype-based information to be able to exploit these traits for future breeding strategies (Chapman et al., 2022). The identification of valuable genetic characteristics relies on the identification of quantitative trait loci (QTLs), development of codominant molecular markers and linkage maps (Oluwole et al., 2021). In Kersting’s groundnut, genome wide association studies (GWAS) identified SNP (single nucleotide polymorphism)-based marker-trait associations related to seed size/weight (Akohoue et al., 2020). GWAS performed on cowpea and castor bean accessions retrieved candidate genes associated with seed size, involved in endosperm/embryo development, and cell elongation (Lo et al., 2019; Xu et al., 2021). Increase in seed size was reported to be associated with enhanced drought tolerance, making this trait very promising in future breeding strategies (Kaydan and Yagmur, 2008; Gholami et al., 2009; Mut and Akay, 2010; Čanak et al., 2020; Martínez-López et al., 2020; Christie et al., 2022). There is also increasing evidence for a cross-talk between drought stress-mediated signaling and endogenous molecular pathways can control the final seeds size (Lv et al., 2019). It has been reported that seed size contributes to drought stress tolerance in camelina (*Camelina sativa* L.), however the response varies depending on biotype and intensity of the imposed stress (Čanak et al., 2020). These findings highlight the need to assess the plant performance under field conditions. Conversely, a larger seed size entails higher management costs by smallholder farmers and increased risk for seed damaging during storage and transport (Ojiewo et al., 2018).

Genes controlling seed dispersal and facilitating harvesting, as well as genes related to seed size may contribute to reduce farmers’ time and efforts. Seed dispersal dynamics allow plant adaptation to climate change by facilitating the migration of species at risk (Fricke et al., 2022). The mutualistic interactions with animals acting as vehicles of dispersal is increasingly affected by the disruptive impact of climate change, and this contributes to further threaten the viability of ecosystems (Fricke et al., 2022). Seed dispersal is facing rapid changes in response to climate change. This trait, resulting from a complex, polygenic genetic background, is still poorly investigated in both crop and model plants (Saastamoinen et al., 2018). In the specific case of crop wild relatives, source of unexplored gene pools for resilience, improved knowledge on this and other seed-related traits is required to better manage long-term conservation (Brǿnnvik and von Wettberg, 2019). Seed yield losses caused by pod dehiscence in forage legumes has been targeted during domestication (Liu et al., 2019; Parker et al., 2021). A better understanding of the mechanisms controlling such a process can help improving the production of forage legumes, particularly those highly resilient species, so far neglected by breeders (Parker et al., 2021; Guo et al., 2022).

Traits that influence the seed nutritional content, the cooking time, and processability have been also identified (Dawson et al., 2019). Climate-resilient crops must be able to provide healthy foods enriched with essential vitamins and minerals (iron, zinc), folate, and fiber. This is typical of neglected species producing highly nutritious seed grains, proxy of food security. In-depth research is necessary, to disclose the properties of such orphan crops and speed up the diversification of healthy diets for the global population (Zenda et al., 2021). Such efforts include the identification of major QTLs, genes and metabolic pathways that shapes the nutritional profile of seeds. This is the case of QTLs controlling iron and zinc concentration in chickpea seeds (Upadhyaya et al., 2016), oil and protein content in soybean (Huang et al., 2020), oil content, protein content, and fatty acids (linoleic and oleic acids) in groundnut (Roorkiwal et al., 2021). Cooking times are influenced by storage, environment, and genotype (Cichy et al., 2019). There is poor knowledge concerning the genetic variation of this trait. Studies carried out using fast and slow cooking bean (*Phaseolus vulgaris* L.) genotypes with different seed types highlighted significant correlations with seed coat thickness and cotyledon cell wall (Bassett et al., 2021). Pasting and cooking properties were assessed in a germplasm collection of orphan legumes that included grass pea (Santos et al., 2018). Multiple seed traits, e.g. size, shape, color, surface influenced physico-chemical parameters as viscosity, hydration capacity and cooking time. A deeper understanding the genetic bases ruling variability across species/accessions could provide useful indicators for the screening of germplasm cooking properties (Santos et al., 2018).

Seed processability is related to the presence of components affecting seed protein digestibility. Such inhibitors, known as antinutrients or antinutritional factors can be removed using dedicated processing technologies, either traditional (cooking, milling, extrusion, germination, fermentation) or innovative technologies (e.g., high-pressure processing, ultrasound, irradiation, microwave). The seed response to these treatments is influenced by genetic factors, such as the qualitative and quantitative profiles of trypsin inhibitors, phytates, tannins, and lectins (Ohanenye et al., 2022). For orphan legumes, the genetic characterization of the most valuable traits and the subsequent breeders’ work are part of a long-term innovation program that must be supported by seed technologists. Once the breeding program has started, several generations are necessary to reach the status of certified seeds. Seed quality changes depending on the seed system, crop species/variety, and location of production.

### 3.3 Seed priming as a tool to mitigate the impact of climate change

Pre-sowing techniques (seed priming) are easily implementable tools, immediately available and applicable to crops, used to increase seed vigor (Pagano et al., 2023a). Seed imbibition is performed under controlled conditions (time, temperature, light/dark) with water, or in solutions containing priming agents, followed by dehydration. Controlled imbibition must be stopped before radicle protrusion occurs, otherwise, seeds will lose desiccation tolerance (Pagano et al., 2023a). Seed priming acts on specific components of the pre-germinative metabolism, a complex array of molecular pathways underlying the seed ability to scavenge the cytotoxic reactive oxygen species and repair DNA damage (Rajjou et al., 2012; Pagano et al., 2023a). The benefits provided by seed priming include improved germination performance and enhanced plant stress tolerance. Seed priming can be performed using water (hydropriming, matrix priming) or a wide range of priming agents that include salts (halopriming), PEG (polyethylene glycol; osmopriming), phytohormones (hormopriming), antioxidant and bioactive molecules (chemical priming), beneficial microorganisms (biopriming) and nanoparticles (nanopriming) (for a more comprehensive description, see review by Pagano et al., 2023a).

Seed priming improves drought tolerance in a wide range of cereal and legume crops (Saha et al., 2022) and several priming agents can effectively promote the seed antioxidant response and osmotic regulation, as mannose (Hameed and Iqbal, 2014), SiO_2_, KNO_3_, salycilic acid (Zheng et al., 2016; Tabassum et al., 2018; Ali et al., 2021), polyethylene glycol (Tounekti et al., 2020). Biopriming mediated by plant growth promoting bacteria can also mitigate drought stress (Pravisya and Jayaram, 2015; Shaffique et al., 2023; Srivastava et al., 2024). Soil salinity was reported to currently affect more than 30% of global irrigated land, being NaCl the most abundant salt in the soil. Seed germination is impaired under salt stress due to unfavorable osmotic potential and ion-mediated toxicity (Khan et al., 2022). Seed priming can alleviate the deleterious effects of salinity, as reported for rice seeds treated with CaCl_2_-based priming (Al-Tamimi et al., 2016). Seed priming applied to wheat using glycine betaine, vitamin B_12_, sodium nitroprusside, jasmonate, CaCl_2_, and KCl enhances chlorophyll content, stabilizes membranes and triggers antioxidant enzymes activity, mitigating salt stress (Sadeghi and Robati, 2015; Keshavarz and Moghadam, 2017).

Heat stress affects the plant physiological and metabolic processes due to protein denaturation, altered membrane integrity, and enzyme inactivation. Hydropriming, osmopriming with CaCl_2_, and hormopriming with salicylic acid were able to support germination of garden pea cultivars under heat stress, improving seed quality and plant development (Tamindžić et al., 2023). Priming with nitrate salts improves nitrate reductase and amylase activity, nitrogen, amino acid and chlorophyll content in leaves, increases proline and sugar content, antioxidant metabolism, under heat stress (Kumar et al., 2021; Tamindžić et al., 2023). Brassinosteroids used as seed priming agents significantly improved the growth and physiological parameters of *Brassica juncea* L. triggering enhanced antioxidant defense under heat stress (Neha et al., 2022). Cytokinins were used as priming agent to assess mitigation protocols against heat stress in wheat (Jaiswal et al., 2022). Field studies showed that plant survival under stress requires proteomic plasticity mediated by the ubiquitin-proteasome system. The effects of flooding stress on seed germination in rice (*Oryza sativa* L.) can be alleviated using seed priming with selenium (Se) (Hu et al., 2022). Selenium could limit malondialdehyde content, increase starch hydrolysis efficiency, enhance antioxidant activities, leading to improved biomass production. However, Se concentrations should be carefully selected in order to avoid the risk of seedling death during long-term flooding (Hu et al., 2022). These treatments also induce molecular mechanisms related to stress memory, accounting for *trans*-generational effects (Louis et al., 2023). Seed priming has an enormous potential to improve seed quality in fragile agroecosystems and fasten the route to food security, promoting drought tolerance and multiple stress resilience. Primed seeds are crucial for successful stand establishment and uniformity when farmers are forced to change the timing of sowing, due to environmental constrains (Singh et al., 2020; Devika et al., 2021; Liu et al., 2022). Improving seed quality support breeders’ work during the selection of ‘climate-smart’ varieties (Pagano et al., 2023a). However, the complexity of seed physiology, genetic background, and the influence of environment result in variability when the technique is applied. Current seed priming technologies would benefit from increased flexibility and efficacy, generated by integrating knowledge in seed biology/technology and agronomy of orphan legumes, moving from bench to field.

### 3.4 From bench to field: ‘on-farm’ seed priming

**Figure 2:**
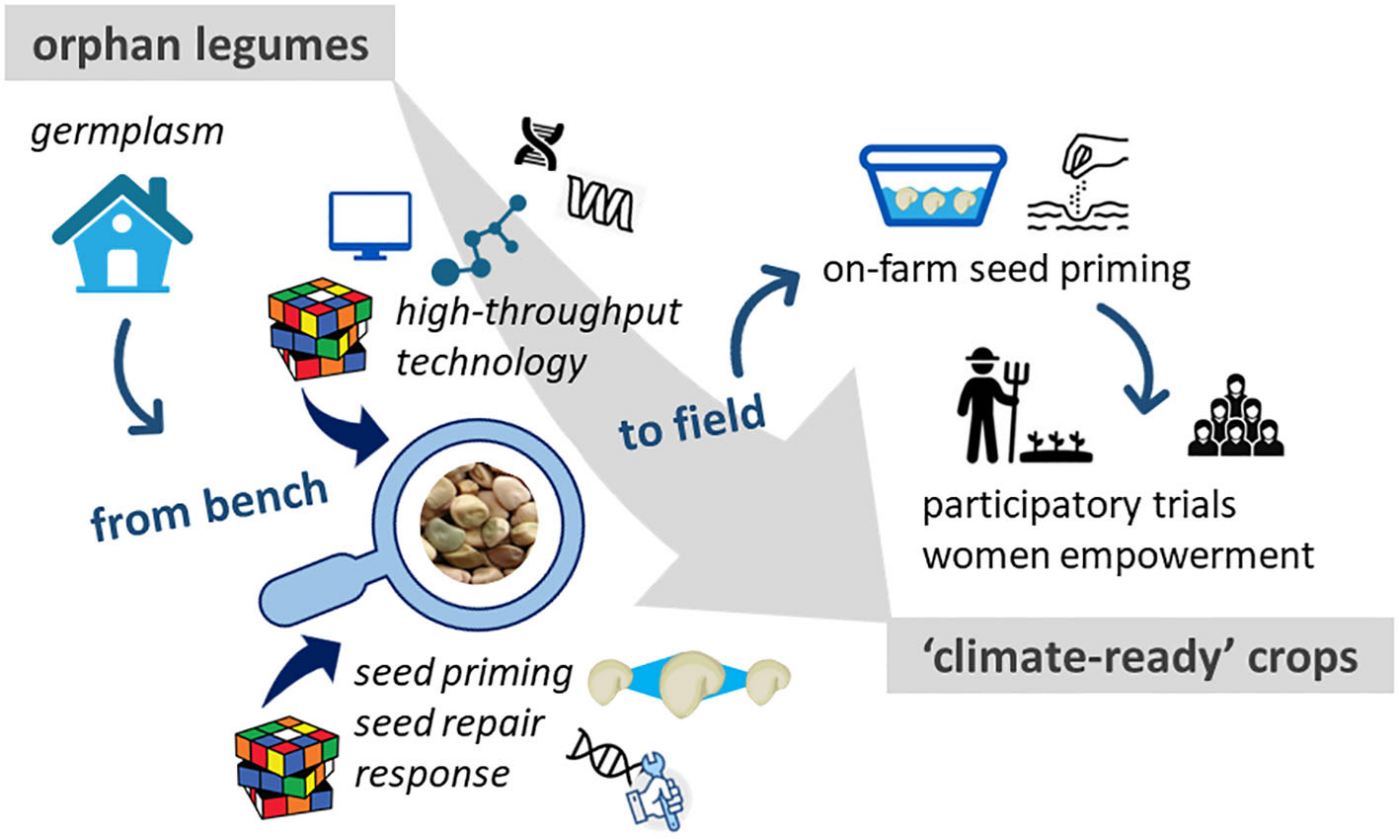
How can orphan legumes reach the status of ‘climate-ready crops’? To what extent can seed quality promote this transition? The ‘seed quality’ perspective, in terms of scientific, technological, and agronomic contributions is depicted. This avenue moves from bench to field as follows. The potential ‘climate-ready’ germplasm available deserves in-depth characterization to assess its real value. This requires the contribution of i) basic research on the molecular mechanisms (e.g. antioxidant players and DNA damage response) underlying seed quality, ii) applied research leading to advanced seed technology (e.g. more effective seed priming protocols), iii) knowledge/technology transfer to farmers and other stakeholders through training, participatory work, with focus on women empowerment.

Seed priming is part of the Seed Treatment Market (estimated size in 2022: > 12.4 billion USD) (Global Market Insights, 2023). Seed companies own patented priming protocols, tailored for major crops but village farmers do not benefit from these resources. ‘On-farm’ seed priming, managed directly by farmers, differs from industrial strategies (Harris et al., 2005; Carrillo-Reche et al., 2018; Devika et al., 2021). In most cases, farmers know that soaking seeds in water before sowing improves plant establishment but they are often unaware of safe limits and drawbacks that might derive from inappropriate treatments. When dealing with fragile ecosystems, the choice of the most suitable priming approach should be carefully handled to maximize benefits. To fully exploit seed priming advantages, specific training with procedures tailored on seed lot/variety should be provided by means of participatory trials with local farmers, seed technologists and researchers (Harris et al., 2005) (**Figure 2**).

## 4 Strategies to enhance seed quality in orphan legumes

Valorization of orphan legumes implies the intensification of their value chains, taking a series of actions ranging from geographic/climatic studies to socio-economic support measures. Multi-actor and cross-disciplinary research networks can bring innovation in orphan legumes production, building novel models in terms of profitability, resilience and environmental sustainability. A recent search carried out in PubMed using the keyword “orphan legume” (https://pubmed.ncbi.nlm.nih.gov/?term=orphan%20legume&timeline=expandedshowed) highlighted a stationary phase in the scientific production in the 1990-2004 period, followed by a progressive increase in the number of articles dealing with this topic, mostly focused on breeding whereas issues of related value chains and socioeconomic impact are still poor investigated. EU-funded, multidisciplinary projects such as CROPDIVA (https://www.cropdiva.eu/), ForEVA (https://www.ecpgr.org/working-groups/grain-legumes/foreva), LEGUMINOSE (https://www.leguminose.eu/), and LEGU-MED (https://www.era-learn.eu/network-information/networks/prima/section-2-call-2019-multi-topic/legumes-in-biodiversity-based-farming-systems-in-mediterranean-basin) are currently promoting the use of underutilized legume species, and the cross-talk between such established research networks will be a powerful driver of more extensive and effective knowledge transfer as well as deeper dialogue with end users.

The case study hereby described is the BENEFIT-Med (Boosting technologies of orphan legumes towards resilient farming systems; https://www.benefit-med.eu/) project, funded by the PRIMA Foundation (https://prima-med.org/), whose main goal is to develop an innovative, and manageable seed priming-based technology for the sustainable production of highly resilient orphan legumes in the Mediterranean Basin.

### 4.1 The BENEFIT-Med project: filling the gap of knowledge

**Figure 3:**
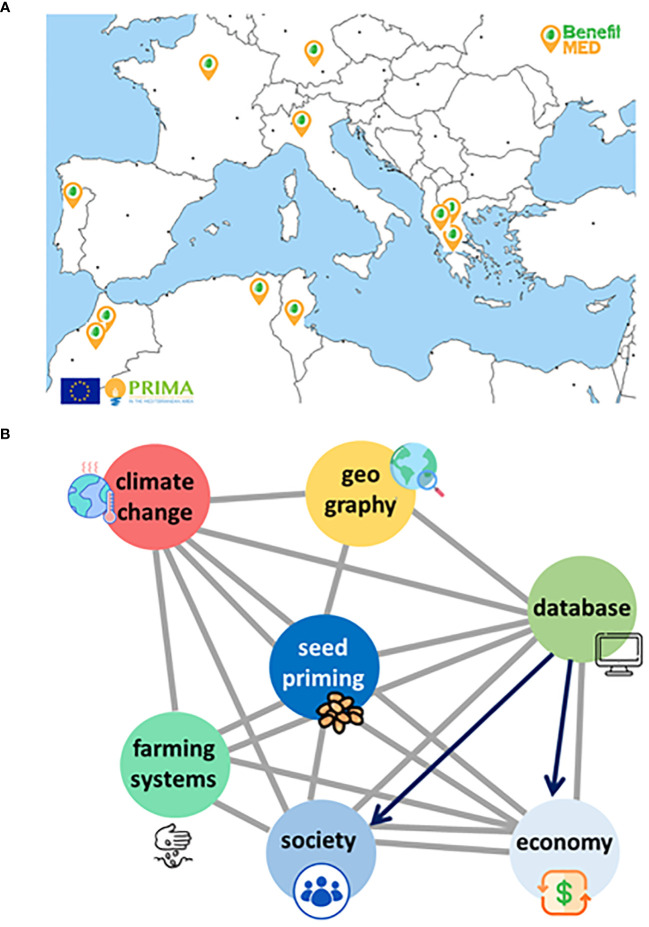
**(A)** The multidisciplinary BENEFIT-Med Consortium across the Mediterranean Basin includes eleven Partners distributed in eight countries: Germany, France, Portugal, Italy, and Greece from the EU side; Morocco, Algeria, and Tunisia from North Africa. **(B)** The ‘seed priming interactome’ represents the multidisciplinary effort and multitarget action of the BENEFIT-Med Consortium, envisaged as a mitigation strategy to boost orphan legumes adoption. Such strategy, relying on dedicated seed technology, requires cross-talk at different levels: i) climate change is targeted through a multidisciplinary approach (e.g. climate science, agronomy), ii) geography is included as mapping the climate vulnerable sites of the Mediterranean area is an essential step for future actions, iii) databases (BENEFIT-Med Hub-Med database) are crucial, as knowledge source and repository, iv) farming systems as the target of seed quality-based innovation, recipients of the benefits brought by orphan legumes, v) socio-economic development of vulnerable regions supported by dedicated actions (dissemination and training, involvement of policy-makers).

Sustainable and resilient agroecosystems are made of agronomic, economic, social and ecological components. The synergic combination of technical and scientific expertise, multi-actors’ networks, and participatory work are required to design such complex structures (Westengen et al., 2023). Trigonella, grass pea, and forage pea are target species selected for valorization by the multidisciplinary BENEFIT-Med consortium, featuring the synergic interaction of eleven partners from eight countries along the Mediterranean Basin (**Figure 3A**). ‘On-farm’ seed priming protocols have been developed at lab scale, and tested under controlled conditions whereas the impact on agronomic performance is currently under evaluation in open-field trials, at different locations in Southern Europe and North Africa (Pagano et al., 2023b). The extreme climate conditions occurred in 2023 allowed to carry out ‘stress-tests’ to assess the benefits of seed priming but at the same time forced the asynchronous experimentation among partner countries. When the output data will be collected, the performance of accession-tailored protocols will be evaluated. The participatory work with local farmers, planned in the near future (2024) will play a crucial role to further optimize treatments and to train farmers about their rationale and effective use of the strategies developed within the BENEFIT-Med consortium. Such tailored seed technologies will be integrated into plant breeding studies to speed up the characterization of pre-breeding materials (Weissmann et al., 2023).

Exploring the tip of the ‘iceberg’ represented by the biodiversity of orphan legumes and transferring such biodiversity into their value chains are crucial. The knowledge of local communities should be preserved and valorized, as a carrier of resilient seed systems (Louwaars and Manicad, 2022; Westengen et al., 2023). It should be also underlined that mining for molecular mechanisms underlying seed quality is an urgent task, being an example of translational research that can accelerate the environmental adaptation of orphan legumes and related agroecosystems (Pagano et al., 2023a). Although seed priming is a main driver in the BENEFIT-Med model, this technology will lose its potential if not integrated into a much wider picture accounting for essential interactors, namely geography, climate, existing farming systems (**Figure 3B**). In BENEFIT-Med, climatologists are also investigating adaptation scenarios to unveil the impact of climate change, estimate site vulnerability and develop adaptation strategies able to support stakeholders and decision-makers (Cradock-Henry et al., 2021).

### 4.2 Vulnerability mapping: an interdisciplinary approach

The rural areas of the Mediterranean region are included in the list of most worldwide vulnerable targets (Ali et al., 2022). In order to support orphan legumes integration into Mediterranean food systems, a deeper understanding of consumption barriers and production constraints is required. Without these efforts, orphan legumes will face extinction because of ‘anthropogenic negligence’ (Talabi et al., 2022). A detailed mapping of vulnerable sites, reflecting the severity of barriers, should be developed. Vulnerability mapping requires an interdisciplinary approach embracing geography, environmental sciences, economics and political sciences, agronomy and climatology. Social aspects integrated with climate exposure metrics will allow to assess vulnerability drivers and adaptation indicators (de Sherbinin et al., 2023; Ngcamu, 2023; Goufa et al., 2024).

Climate-vulnerable sites are mapped based on socio-cultural barriers resulting from the different sensitivity and adaptation response of individuals and specific social/cultural groups. Economic barriers impair seed production and seed systems development, compromising market competitiveness. The challenge lies in promoting financial investments in order to strengthen the role of orphan legumes in the sustainable agrosystems of the Mediterranean region. Economic failure is the result of weak/missing supportive policies (political barriers), agriculture-related policies and policy frameworks (institutional barriers) should be revised to expand the use of orphan legumes e.g., by improving services to rural populations, adding these species in the national export strategy, or investing in the maintenance of traditional knowledge (Aktürk and Dastgerdi, 2021). Regular cross-talks with stakeholders are absolutely required to stimulate continuous participatory work and mitigate conflicting interests (Aktürk and Dastgerdi, 2021). Furthermore, awareness on climate-change impact and the potential of orphan legumes as climate-smart crops, as well as the nutritional value of traditional dishes, can be raised through education. Initiatives such as the CGIAR Generation Challenge Program (https://www.generationcp.org/) have contributed to build a new generation of breeders who vehicle cultural changes. Multiple socioeconomic factors (gender, class, ethnicity, age, disability) contribute to women vulnerability in rural areas (Abu-Rabia-Queder and Morris, 2018; FAO, 2023). Prolonged and high workload limits the access of young girls, in charge of water collection, to school. This compromises the possibility to bridge the gender gap in education, among the Sustainable Developmental Goals (SDGs) targets (UNICEF, 2020; Hamlet et al., 2021). Furthermore, climate-driven migration disrupts communities, charging women with heavier responsibilities and economic insecurity (Nwoke and Ibe, 2014; FAO, 2023).

Vulnerability mapping across the Mediterranean basin will face these problems, attempting to provide an in-depth description of the socioeconomic and climate scenarios essential for understanding the dynamics involving human/climatic factors and for the prediction of future expected impacts. The multidisciplinary character of the BENEFIT-Med team will lead to a dedicated vulnerability mapping of the Greater Mediterranean Region (GMR), as a starting point to develop more effective roadmaps focused on the mitigation effects resulting from the productive introduction of target species.

### 4.3 The Mediterranean region: a climate-change hot-spot

The Mediterranean region is considered a climate-change hot-spot of the 21^st^ century (Giorgi, 2006). In this area, global warming rates are occurring at a greater pace and temperature extremes are expected to be reached (Lionello and Scarascia, 2018). Global air temperature increases by 1.5 and 2.0°C might lead to an increase of maximum air temperatures in the order of 2.2 and 3.0°C, respectively. Projected warming rates are connected to increased heat wave intensities and more frequent heat waves (Perkins-Kirkpatrick and Gibson, 2017), leading to reduced soil moisture and increased water stress for crops during all seasons, but with larger reduction rates during winter and spring, the main planting season for orphan legumes in the GMR (Samaniego et al., 2018). The evolution of new farming systems across the GMR, based on reliable climate information, is inevitable. One of the major challenges to overcome is the identification of the impact of climate change and variability on existing and new farming systems.

A suite of regional climate projections already exists from the Coordinated Regional Climate Downscaling Experiment (CORDEX) initiative (https://cordex.org/). Through global partnerships in regional climate modelling, various regional climate models (RCMs) are driven by large-scale general circulation models (GCMs) to derive regional-scale climate information under different emission scenarios. However, these climate projections are associated with large uncertainties as well as systematic biases, which should be reduced before applying them to climate change impact assessments (Laux et al., 2021). For the quantification of devastating extreme events in the GMR, such as the joint occurrence of severe drought and heat events, more sophisticated (multivariate) methods for bias correction (BC) are necessary (Adeyeri et al., 2023). Based on BC approaches, the co-variability of the most critical climate variables can be retained (Dieng et al., 2022; Olschewski et al., 2023), allowing the mapping of crop suitability and vulnerability to climate risk under different climate change scenarios. Crop suitability mapping is based on the growing thresholds for crops in relation to climate conditions. Within the BENEFIT-Med project, the concept of climatic crop suitability will be extended by edaphic, hydrological, and topographic conditions. Suitable threshold parameters for specific orphan legumes will be derived from field experimentations and finally upscaled to the GMR. Crop vulnerability, in this context, will be analyzed through the characterization of current and future patterns in soil temperature and soil moisture. In fact, soil temperature and soil moisture, besides other factors, affect seed germination and govern stand establishment (Elias et al., 2019).

### 4.4 Organization of the available knowledge

The current knowledge on orphan legumes is still fragmented, dispersed across repositories, from which data on few species can be retrieved. This is the case of *Moringa oleifera* L. and *Vigna subterranea* L., whose genomes are accessible at ORCAE-AOCC (Online Resource for Community Annotation of Eukaryotes - African Orphan Crops Consortium) (https://bioinformatics.psb.ugent.be/orcae/aocc/), a public genome annotation resource dedicated to underutilized crops, designed to facilitate stakeholders’ efforts (Yssel et al., 2019). Ideally, a globally accessible database that collects all the information on orphan legumes should be assembled and elaborated for working at multiple levels: as interface for interactions with public authorities, for dissemination/scientific/technical activities. A data model spanning the main components of the crop value chain in the food system has been suggested by Nizar et al. (2021). Such initiatives should be expanded to orphan legumes, merging the knowledge collected within past and current research projects.

## 5 Conclusions

This review highlights the drawbacks that must be overcome before orphan legumes could reach the state of ‘climate-ready crops’. The technical/social interfaces at which multiple actors play - still inadequate - roles are discussed. From the technical side, engagement of orphan legumes in climate-resilience breeding has become a priority and breeders can benefit from the current multidisciplinary approaches and high-throughput technologies to address the need of high-quality seeds. As for seed priming, an ideal roadmap should move from basic research to the translational and agricultural research domains, fishing new hallmarks of the seed response from the complex molecular pathways that showcase the pre-germinative metabolism before their validation in applied purposes. Seed systems should be reinforced by balancing the increased potential for seed quality and profitability offered by formal systems and an easier access to seeds should be ensured by community-based seed systems.

## Author contributions

AlB: Writing – review & editing, Writing – original draft, Conceptualization. CC: Writing – review & editing. AM: Writing – review & editing. AP: Writing – review & editing. PL: Writing – review & editing. HM: Writing – review & editing. LR: Writing – review & editing. ET: Writing – review & editing. DC: Writing – review & editing. CK: Writing – review & editing. LG: Writing – review & editing. SG: Writing – review & editing. SM: Writing – review & editing. AE: Writing – review & editing. MH: Writing – review & editing. BK: Writing – review & editing. HE: Writing – review & editing. JN: Writing – review & editing. SA: Writing – review & editing. AzB: Writing – review & editing. MB: Writing – review & editing.
